# TreatmENT of AnastomotiC LeakagE after colon cancer resection: the TENTACLE – Colon study

**DOI:** 10.1186/s12893-025-02954-1

**Published:** 2025-05-15

**Authors:** Jobbe M. G. Lemmens, Sander Ubels, Nynke G. Greijdanus, Kiedo Wienholts, Marleen M. H. J. van Gelder, Albert Wolthuis, Jérémie H. Lefevre, Kilian Brown, Matteo Frasson, Nicolas Rotholtz, Quentin Denost, Rodrigo O. Perez, Tsuyoshi Konishi, Martin Rutegård, Susan L. Gearhart, Thomas Pinkney, Muhammed Elhadi, Roel Hompes, Pieter J. Tanis, Johannes H. W. de Wilt

**Affiliations:** 1 Department of Surgery, Radboud University Medical Centre, Radboud Institute for Health Sciences, Nijmegen, the Netherlands; 2 Department of Surgery, Canisius Wilhelmina Hospital, Nijmegen, the Netherlands; 3 Department of Surgery, Amsterdam University Medical Centres, University of Amsterdam, Amsterdam, The Netherlands; 4 Cancer Centre Amsterdam, Treatment and Quality of Life, Amsterdam, The Netherlands; 5 Cancer Centre Amsterdam, Imaging and Biomarkers, Amsterdam, The Netherlands; 6 IQ Health Science Department, Radboud University Medical Centre, Nijmegen, the Netherlands; 7 Department of Surgery, UZ Leuven, Leuven, Belgium; 8 Department of Digestive Surgery, Sorbonne Université, AP-HP, Hôpital Saint Antoine, Paris, France; 9 Department of Colorectal Surgery, Royal Prince Alfred Hospital, Sydney, Australia; 10 Department of Surgery, Hospital La Fe, University of Valencia, Valencia, Spain; 11 Department of Surgery, Hospital Alemán, Buenos Aires, Argentina; 12 Bordeaux Colorectal Institute, Clinique Tivoli, Bordeaux, France; 13 Colorectal Surgery, Hospital Alemão Oswaldo Cruz, São Paulo, Brazil; 14 Department of Surgery, Hospital Beneficência Portuguesa de São Paulo, São Paulo, Brazil; 15 Department of Colon and Rectal Surgery, The University of Texas MD Anderson Cancer Center, Anderson, Texas, USA; 16 Diagnostics and Intervention, Surgery, Umeå University, Umeå, Sweden; 17 Department of Surgery, Johns Hopkins University School of Medicine, Baltimore, Maryland USA; 18 Academic Department of Surgery, University of Birmingham, Birmingham, UK; 19 Faculty of Medicine, University of Tripoli, Tripoli, Libya; 20 Department of Oncological and Gastrointestinal Surgery, Erasmus Medical Centre, Rotterdam, the Netherlands

**Keywords:** Treatment, Severity, Anastomotic leakage, Colon cancer resection

## Abstract

**Background:**

Anastomotic leakage (AL) is a common and severe complication after colon cancer resection, but studies investigating various treatment strategies and factors influencing outcomes are scarce.

**Objectives:**

(1) To identify predictive factors associated with 90-day mortality and 90-day Clavien-Dindo grade 4–5 complications amongst patients who developed AL following colon cancer resection with subsequent development and validation of prediction models, and (2) to explore and compare the effectiveness of various treatment strategies for AL following colon cancer resection, adjusting for type of index surgery, different leak entities and patient factors.

**Methods:**

The TENTACLE – Colon is an international multicentre retrospective cohort study. Consecutive patients with AL after colon cancer resection operated between 1 January 2018 and 31 December 2022 from participating centres will be included. The planned sample size is 2000 patients. The primary outcome is 90-day mortality and the co-primary composite endpoint is Clavien-Dindo grade 4–5 complications. Secondary outcomes include: hospital and intensive care unit length of stay, number of radiological and surgical reinterventions within one year after resection, mortality (in-hospital, 30-day, and 1-year), the comprehensive complication index, and 1-year stoma-free survival. For objective 1, regression models will be used to identify predictors associated with 90-day mortality and grade 4–5 complications. For objective 2, comparative analyses of various treatment strategies will be performed for the specified outcomes, adjusting for patient, tumour, resection and leakage characteristics.

**Trial registration:**

This study is registered at clinicaltrials.gov (NCT 06528054) since July 30th, 2024.

## Introduction

Anastomotic leakage (AL) is a common and severe complication after colon cancer resection. Recent studies report leak rates between 4.5% and 8.4%, despite ongoing improvements in perioperative care and surgical techniques. [1–6] AL is associated with re-intervention(s), prolonged hospital stay, a higher likelihood of permanent stomas, and mortality. [7–13] Numerous population-based studies have shown that postoperative mortality rates vary from 12 to 19% among colon cancer patients with AL. [1, 14, 15] Long-term consequences of AL include omission of adjuvant chemotherapy and a possible stage-dependent reduction in survival. [16, 17]. Despite these alarming numbers, comparative studies evaluating different treatment strategies for AL are lacking.

In current clinical practice, treatment decision-making is frequently based on the clinical presentation, resource availability, surgeon preference and experience, and leakage characteristics. Such leakage characteristics may include defect size and location, presence of ischemia, tension on the anastomosis, presence of a localized abscess, purulent peritonitis, faecal contamination, and sepsis. [18] However, little is known regarding these leakage characteristics, and to what extent these factors contribute to treatment decision-making and influence postoperative outcomes.

Current treatment options for AL include conservative management with antibiotic regimens, surgical interventions (e.g. dismantling of the anastomosis), radiological drainage, endoscopic clipping, or a combination of these modalities. [19–22] Evidence regarding effectiveness of these treatment modalities remains scarce, and identifying the optimal treatment modality individualized for a specific patient with specific characteristics could improve patient outcomes.

Therefore, this study has two main aims: (1) to identify predictive factors associated with 90-day mortality and 90-day Clavien-Dindo grade 4–5 complications amongst patients who developed AL following colon cancer resection and to develop and validate prediction models for these two main outcomes and (2) to explore and compare the effectiveness of various treatment strategies for AL following colon cancer resection, adjusting for type of index surgery, different leak entities and patient factors.

## Methods

### Study design

**Fig. 1:**
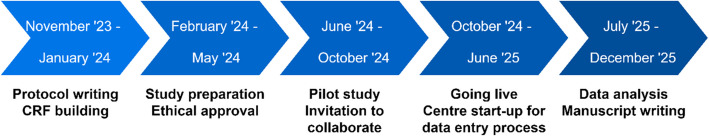
TENTACLE – Colon study timeline

The TENTACLE – Colon is an international multicentre retrospective cohort study in which all consecutive patients who developed AL after colon cancer resection operated between 1 January 2018 and 31 December 2022 will be included from each participating centre. Last date of follow-up of included patients will be registered. Data collection started in October 2024 and recruitment will continue until June 2025. See Fig. 1 for the timeline.

The TENTACLE—Colon study is open for participation by all centres worldwide performing colon cancer resections, irrespective of geographic, economic or institutional setting. The TENTACLE — Colon study will be disseminated across multiple (inter)national surgical societies to increase the number of inclusions and optimize the probability of obtaining robust results. The current study is an investigator-initiated study without financial support.

### Study population

Inclusion criteria are: (1) aged 18 years or older, (2) surgical resection for primary colon cancer (cT1-4b, N0-2, M0-1, colon cancer was defined as when the lower border of the colon cancer was above the level of the sigmoid take-off on imaging) with formation of a primary colonic anastomosis above the peritoneal reflection, with or without a diverting stoma, and (3) postoperative AL defined as: “any clinical, radiological or intraoperative sign(s) of disrupted integrity of the colonic anastomosis (i.e. ileo-colic, colo-colic or high colo-rectal anastomosis (based on surgery report) and/or leakage from a blind loop of the colonic anastomosis. [23] This also includes suspected leaks with any degree of extraluminal air or fluid on computed tomography (CT) scan, perianastomotic abscess, purulent peritonitis without clear anastomotic defect, or any other suspicious condition in which there is no ultimate macroscopic proof of disrupted anastomosis.”

Regarding the type of colon cancer resection, the following patients will also fulfil the inclusion criteria: patients who underwent cytoreductive surgery simultaneous with resection of the primary colon cancer with or without hyperthermic intraperitoneal chemotherapy, simultaneous ablations/resections of metastasis, multivisceral resection, emergency resection, patients diagnosed with perforated disease, peritumoural abscess and/or fistula, and acute obstructions.

Exclusion criteria are (1) surgical resection for benign disease, (2) surgical resection for recurrent colon cancer, (3) diagnosis of any primary colon malignancy other than adenocarcinoma (e.g. neuroendocrine tumour, gastro-intestinal stromal tumour), and (3) any clinical condition that does not fulfil the broad definition of AL as used in this study (e.g. only free air on CT scan that is considered to be compatible with a certain postoperative day in the absence of any other clinical signs related to a potential anastomotic leakage).

### Study parameters

#### Hospital characteristics

Upon study entry, each participating centre will be required to complete a questionnaire to gather comprehensive information regarding institutional characteristics. This questionnaire covers the following subjects: hospital type, annual volume of colon cancer resections, estimated anastomotic leakage rate, number of hospital beds and availability of hospital resources, protocols, and diagnostic and treatment modalities.

#### Patient and tumour characteristics

The following patient characteristics will be collected: sex, age, height, weight, body mass index, Charlson Comorbidity Index, American Society of Anaesthesiologists classification, smoking status, baseline hemoglobin level and history of immunosuppressive medication. The following tumour characteristics will be collected: histology, location, pathological T-, N- and M-stage according to the Union for International Cancer Control classification, preoperative tumour-related complications, surgical interventions before index colon cancer resection, preoperative anaemia treatment, and neoadjuvant treatment.

#### Surgical and intraoperative characteristics

The following characteristics regarding preparation before colon cancer resection will be registered: setting of resection, preoperative antibiotic and mechanical bowel preparation use. The following surgical characteristics will be collected: date of resection, start and end time of resection, intention of resection, abdominal approach, conversion, type of resection, multivisceral resection, other simultaneous intervention applied, number and type(s) of anastomosis, configuration of colonic anastomosis, problems during anastomosis construction, stoma creation, indocyanine green assessment, intraoperative complications, blood loss during resection, and amount of blood transfusions.

#### Diagnostic characteristics

Collected diagnostic characteristics will be: date and time of diagnosis of AL, clinical setting at time of leak diagnosis, antibiotics and nasogastric tube use, modalities applied to assess anastomotic integrity, and clinical, biochemical and intensive care unit parameters. The following leakage characteristics at diagnosis will be collected: radiological characteristics, intraoperative characteristics, location and estimated circumference.

#### Treatment of anastomotic leakage

Details of all therapeutic modalities and strategies utilized will be collected, including primary, secondary, tertiary, and quarternary treatments: date and time of start treatment, need for re-admission, and (specification of) applied radiological, endoscopic, and surgical interventions.

#### Follow-up of treatment

The following characteristics will be collected to assess outcomes after leakage treatment: leak healing and modality that confirmed leak healing, date of initial hospital discharge, total length of intensive care unit stay, date of death (if applicable), recurrent colon cancer, stoma status, total number of radiological and surgical interventions within one year after colon cancer resection, adjuvant chemotherapy, and diagnosis of other complications with corresponding grading of severity. [24].

### Outcome measures

The primary outcome is 90-day mortality, and the co-primary outcome is a composite endpoint of 90-day Clavien-Dindo grade 4–5 complications. [24] Secondary outcomes include: length of hospital stay, length of intensive care unit stay, number of radiological and surgical reinterventions within one year after resection, mortality (in-hospital, 30-day, and 1 year), comprehensive complications index, and 1-year stoma-free survival. [25].

### Sample size calculation

This study is exploratory in nature, and its primary objective is to utilize a large detailed dataset to examine to what extent specific factors of AL are associated with the severity of the leakage and the impact of various treatment strategies on primary and secondary outcome parameters, with adjustment for relevant covariates.

To establish a risk score comprising at least 12 candidate predictors, with a 10% incidence of 90-day mortality amongst AL patients following colon cancer resection and a root mean square percentage error of 5%, it is necessary to include 680 patients with AL. [26] However, to ensure robustness, to enable the development and validation of an evidence-based prediction model, and to create a solid foundation for future research related to AL, the aim is to include at least 2000 patients with AL following colon cancer resection.

### Pilot study

After the study protocol and online case report file (CRF) were developed, the international steering committee consisting of 15 experts on AL after colon cancer resection working across 11 different countries participated in the pilot test (Table 1). During pilot testing, the experts were asked to include at least 5 patients into the online CRF and to provide feedback on the protocol and online CRF. The feedback was evaluated by the study group, and used to refine the protocol and CRF in order to meet international standards and clarity on definitions and the use of the online CRF.

**Table 1:** International steering committee TENTACLE – Colon study

Member		Hospital	City	Country
1	Hans de Wilt	Radboudumc	Nijmegen	The Netherlands
2	Pieter Tanis	ErasmusMC	Rotterdam	The Netherlands
3	Roel Hompes	Amsterdamumc	Amsterdam	The Netherlands
4	Albert Wolthuis	UZ Leuven	Leuven	Belgium
5	Kilian Brown	Royal Prince Alfred Hospital	Sydney	Australia
6	Jérémie Lefevre	Sorbonne Université, AP-HP, Hôpital Saint Antoine	Paris	France
7	Martin Rutegård	Umeå University	Umeå	Sweden
8	Quentin Denost	Clinique Tivoli	Bordeaux	France
9	Nicolas Rotholtz	Hospital Alemán	Buenos Aires	Argentina
10	Thomas Pinkney	University of Birmingham	Birmingham	United Kingdom
11	Rodrigo Perez	Hospital Alemão Oswaldo Cruz	São Paulo	Brazil
12	Susan Gearhart	Johns Hopkins Medicine	Baltimore	United States of America
13	Tsuyoshi Konishi	The University of Texas MD Anderson Cancer Center	Anderson	United States of America
14	Matteo Frasson	Valencia University Hospital La Fe	Valencia	Spain
15	Muhammed Elhadi	Faculty of Medicine, University of Tripoli	Tripoli	Libya

### Data handling and regulatory considerations

The Castor database system (https://www.castoredc.com/) will be used to collect data through online CRFs. Castor is an officially certified online medical research database system that fully complies with all pertinent regulations (i.e. GDPR, HIPAA, ICH-GCP, ISO 27001, ISO 9001, FDA 21 CFR part 11) and strictly adheres to international security standards. The CRF contains information points, definitions, and guidelines to facilitate accurate scoring of the specified parameters. The data entered will exclusively be visible to collaborators from the designated hospital, and access to the complete study database will be limited to coordinating investigators and principal investigators.

All pseudonymized patient data will be entered by or under supervision of the treating physician(s). Each patient will be coded with a unique patient number before being entered into the database. The physician will keep a password-protected file that can identify individual patients, which will be locked away in their practice. This file can be accessed by the local investigators if needed, for example in case a relevant new research question requires entry of additional data into the database.

All (up to 4) collaborators from the participating centres will receive an invitation to the Castor database along with step-by-step manuals and an online training module to ensure homogeneity in the inclusion process and during data entry, and to ensure that all eligible patients with AL within 90 days after colon cancer resection will be screened.

### Data cleaning, verification and validation

After a participating centre has finished data entry, the coordinating investigator will initiate data cleaning, verification and validation procedures to improve data completeness and to assess data quality and case ascertainment. Data cleaning will involve the use of algorithms to scrutinize the data for any missing values, data inconsistencies and typographical errors. All issues identified will be communicated to the respective local investigators enabling them to verify, adjust, or add the (missing) data. For data validation, data accuracy and case ascertainment will be assessed. Data accuracy will be assessed by cross-checking data recorded in Castor with medical records by a local independent validator. At random, 20% of the participating centres will be selected for data validation, wherein a subset of predefined key parameters of 10–20% of the inclusions of a centre will be validated. Case ascertainment will be assessed to identify a systematic difference in the selection of patients per centre, defined as the proportion of included cases compared to the total amount of eligible cases. To assess case ascertainment, collaborators will be asked whether they entered data of all consecutive patients with an AL within the study period, or whether a sample was included (and if so; for which period). Furthermore, participating centres will be asked about their annual number of colon cancer resections. The AL rate was conservatively estimated at 3% for all years within the study period. [27] If the number of included cases is lower than the estimated number of cases, the collaborators will be asked to substantiate this discrepancy. For all procedures, a step-by-step manual as well as additional support will be available.

### Data availability

Data may be available upon reasonable request. Only collaborators with appropriate qualifications and pertinent research inquiries are eligible to request access to the data. The principal investigators of the TENTACLE – Colon study will assess the relevance and appropriateness of the request and their verdict is decisive. If data will be transferred, it will only be conducted under the appropriate ethical and data transfer agreements.

### Statistical analysis

Statistical protocols have been drafted with an experienced biostatistician (M.v.G). These analytical strategies are in line with the previous TENTACLE – Esophagus and TENTACLE – Rectum projects. [18, 28–30].

#### Main study objective 1

The goal of this study is to identify predictive factors associated with 90-day mortality and the co-primary composite endpoint Clavien Dindo grade 4–5 complications among patients who developed AL following colon cancer resection. Subsequently, two distinct prediction models for these two endpoints will be developed separately with several clinically relevant patient, tumour, resection, diagnostic and leakage characteristics, and internally validated. The development and validation of the prediction models will be in agreement with the transparent reporting of a multivariable prediction model for individual prognosis or diagnosis guidelines. [31] First, univariate analysis will be performed on parameters that are considered to be potentially relevant. These parameters will be entered into separate binary logistic regression models with 90-day mortality and Clavien Dindo grade 4–5 as outcome parameters in order to explore associations in the data. Second, relevant parameters that are also considered to be clinically relevant based on literature and/or expert opinion will be selected for multivariate analysis. Backwards stepwise selection will be used to exclude variables with P values > 0.05 from the model. Results will be presented as odds ratio with 95% confidence intervals (CI). A 2-tailed p < 0.05 will be considered statistically significant. Third, the multivariable models will be internally validated by bootstrapping, using 5000 bootstrap resamples. Finally, the models will be created based on the final bootstrapped multivariable regression analysis. Each model will be separately incorporated into an online tool and will be made available at https://www.tentaclestudy.com/.

If the casemix is found to be strongly associated with outcome relative to the mortality score (to the extent that the mortality score is of limited value in the regression model), latent class analysis will be used. [32] The parameters used for the mortality score will be used to create casemix-corrected classes of AL severity.

Sensitivity analysis will be performed in subgroups of patients undergoing various types of colectomies, and for different settings (e.g. elective, emergency) of colon cancer resection. These analyses will investigate whether the obtained model is useful for all types and settings of colon cancer resection. If substantial differences are observed between the initial and subgroup analyses, the possibility of composing different scoring systems will be considered.

#### Main study objective 2

The second main objective is to explore and compare the effectiveness of various treatment strategies for AL following colon cancer resection, adjusting for patient, tumour, resection and leakage characteristics.

Relevant treatment parameters identified in the first analysis will be considered independent variables in this analysis. The association between characteristics of colon cancer resection, AL, and outcome measures (e.g. 90-day mortality) will be evaluated for the exposures in regression analysis. Where appropriate, statistical adjustment using propensity score matching considering patient, tumour, resection, and/or leakage characteristics, as well as timing, potential delay and clinical setting of AL diagnosis will be performed to account (potential) confounding.

Based on the results of this analysis, subgroups of patients will be created based on individual resection or leakage characteristics (or a combination of these). The anticipated treatment strategies to be compared are conservative treatment, surgery with preservation of the anastomosis and surgery with dismantling of the anastomosis with performing an end-ostomy. The efficacy of AL treatment strategies will be assessed in regression models for the different outcome parameters and adjusted for patient, tumour, resection and leakage characteristics, where appropriate. All analyses for objective 2 will be conducted using modified Poisson regression, with results expressed as risk ratios with corresponding 95% confidence intervals.

### Ethical considerations

This study will be conducted in compliance with the principles of the declaration of Helsinki. The medical ethical committee of the Radboud university medical centre in Nijmegen, The Netherlands, has thoroughly reviewed all documents, including the study protocol and granted approval (file number: 2024–17491). The need for central informed consent was waived, but local ethical requirements may differ per geographical region, country, or hospital. The study protocol and relevant documents will be provided to all participating centres in case the centres need local ethical approval. The TENTACLE – Colon study is registered on Clinicaltrial.gov (NCT 06528054). The study protocol and the letter of ethical approval is also available on the website: https://www.tentaclestudy.com/.

### Publications

The TENTACLE study team aims to publish two main manuscripts covering the main objectives. These manuscripts will be submitted to peer-reviewed journals. All members of the international steering committee are fully involved in conducting this study and will be included as (co-)authors in the publications. All (up to) 4 collaborators and local independent validator (if applicable) will be granted (PubMed-citable) corporate authorship under the name ‘the TENTACLE – Colon Collaborative Group’ in all manuscripts using data of the TENTACLE – Colon study. The affiliation information of the collaborators will be the hospital whose patients were included.

### Study status

Halfway through the inclusion period, a total of 315 centres from 56 countries across 6 continents are collaborating, resulting in the enrolment of 2506 patients.

## Discussion

Anastomotic leakage (AL) remains a common and severe complication after colon cancer resection. Most research to date has focused on the prevention and prediction of AL, but large comparative studies evaluating various treatment modalities for AL are scarce. [33–35] As a result, the optimal treatment of AL after colon cancer resection and the subsequent important clinical outcomes remain largely unknown. There are several explanations for this knowledge gap. Treatment of AL is generally chosen on a case-by-case basis, depending on several patient and resection characteristics, and is influenced by the preferences and expertise of the treating surgeon. More specifically, AL is a heterogeneous and highly complex complication, which is likely to hamper the initiation of standardized institutional treatment protocols and the design of prospective studies. Furthermore, the diverse clinical presentation and wide variety of treatment approaches complicates the interpretation and generalizability of small underpowered studies.

The TENTACLE – Colon study aims to address this knowledge gap and will therefore provide valuable insights into the severity and treatment of AL after colon cancer resection. This global effort, combined with detailed data collection, will facilitate robust (comparative) analyses and the results can provide a solid basis for evidence-based and personalized treatment decisions. The international success of the previous TENTACLE studies in esophageal and rectal cancer surgery underscores the relevance, feasibility and importance of the current study. [18, 28, 29, 36].

The main strengths of the present study are the high level of detail of the data collected and the large number of planned patient inclusions. This large cohort is needed to perform regression analyses with various patient, tumour, resection and leakage characteristics to explore and identify distinct clinical presentations of colonic AL. For each clinical presentation, relevant subgroup analyses can be performed to compare relevant treatment strategies. The inclusion of a large cohort is feasible due to the international collaborative design of the current study, which increases the generalizability of findings across various populations. A pilot study was conducted among core collaborators from diverse continents to ensure that the online CRF includes parameters relevant to various geographical regions along with clear definitions.

The main limitation is the retrospective design of the study. A prospective design was not considered feasible due to logistical reasons and the need to include a large cohort, while AL occurs in no more than 8.4% of patients. (1) However, the results of this study will be hypothesis generating, and this knowledge can be used to guide the design process of future prospective studies. Another limitation is the risk of confounding by indication, as patients receiving certain treatments may be inherently different from those receiving other treatments. In this study, regression analysis using sophisticated statistical techniques to account for confounding of detailed data within a large cohort should mitigate much of this expected confounding. However, residual confounding may occur if the regression analysis cannot adequately adjust for all confounding factors. Finally, given the international and collaborative nature of the study, there is a risk of selection bias as not all centres worldwide will be collaborating. This may bias the dataset towards patients from more scientifically invested centres, which may limit generalizability to some extent.

For current clinical practice, the results of the TENTACLE – Colon study could have an impact on management of AL. The prediction model (i.e. the first main objective) will indicate the severity of AL in terms of mortality by considering patient, tumour, resection and leakage characteristics. Clinicians can use this model to distinguish patients at high risk of mortality from those at low risk. This is useful because the severity of AL is nowadays classified by how it is treated, but this classification can, by definition, not be used to prospectively guide clinical decision-making. [24] Furthermore, the evaluation of treatment strategies (i.e. the second main objective) for different subgroups of AL might indicate the best treatment strategy for each distinct clinical presentation. Currently, patients can be treated with a wide range of treatment modalities, mainly due to the lack of evidence to support a specific modality for a specific clinical presentation of AL. Therefore, clinicians can use the prediction model and insights from the treatment evaluations of different clinical presentations to faciliate evidence-based and personalized treatment decision-making when AL is diagnosed.

## Data Availability

Data may be available upon reasonable request.

## Abbreviations

AL: Anastomotic leakage.
CT: Computed tomography.
CRF: Case report file
